# Changing Technology Use, Confidence, and Support Needs Among Older Adults in United Kingdom Retirement Villages: Mixed Methods Study

**DOI:** 10.2196/92816

**Published:** 2026-08-31

**Authors:** Angela Higgins, Steve Benford, Michelle Hall, Holly Blake, Kevan Murray, Praminda Caleb-Solly

**Affiliations:** 1 School of Computer Science, University of Nottingham, Wollaton Road, Nottingham, England, NG8 1BB, United Kingdom, 44 115 951 4251; 2 School of Health Sciences, University of Nottingham, Nottingham, England, United Kingdom; 3 NIHR Nottingham Biomedical Research Centre, Nottingham, England, United Kingdom; 4 Nottingham Trent University, Nottingham, Nottinghamshire, United Kingdom

**Keywords:** older adults, technology adoption, assistive technology, older adult, technology use

## Abstract

**Background:**

Assistive technologies can support independent living among older adults, helping manage health and maintain independence, but uptake is often constrained by attitudes, confidence, and socioeconomic factors. The COVID-19 lockdowns accelerated technology use across all age groups, offering a natural experiment to examine changes in adoption.

**Objective:**

This study aimed to examine changing patterns of technology use in older adults, to provide insight as to how service providers can support the use of technology to enhance resident independence and well-being.

**Methods:**

Two cross-sectional surveys were conducted in United Kingdom retirement villages operated by a single housing provider, one immediately before the pandemic (2020, n=1707 across 19 sites) and one after lockdowns were lifted (2023, n=45 at one site), assessing technology confidence, device ownership, internet use, and learning pathways. Semistructured interviews with 8 participants in a technology trial scheme provided qualitative depth. Integration followed a connecting approach, with quantitative findings identifying patterns and qualitative data contextualizing them.

**Results:**

Technology adoption increased significantly between 2020 and 2023, with older adults reporting greater confidence (*M*=2.29 to *M*=3.36; *P*<.001), higher device ownership, and increased internet use across all activities. Self-education and informal support from family or friends were the most common pathways to adoption, with 69% (31/45) of 2023 participants reporting learning from friends or family compared with 20% (44/216)‐28% (416/1491) in 2020. Age-related differences in confidence observed in 2020 were no longer apparent in 2023, although gender disparities persisted across all surveys, with men reporting significantly higher confidence (*P*=.007, *r*=0.42 in 2023). No significant differences were found based on socioeconomic indicators. Qualitative findings revealed that positive technology experiences were predominantly social in nature, while health-related features such as medication reminders went largely unused. Integration of the 2 strands showed that the qualitative data expanded, confirmed, or explained the survey findings, while the gender gap remained unresolved by the qualitative strand due to the self-selected nature of the interview sample.

**Conclusions:**

Findings demonstrate that the pandemic accelerated lasting increases in technology adoption among older adults, though these changes also reflect broader societal, technological, and community-level factors. The persistent gender gap in confidence likely reflects cumulative life-course disadvantages in workplace technology exposure. Technology provision for older adults appears most effective when embedded within the social fabric of a community. These results provide evidence for housing providers and policymakers to formalize peer support pathways, introduce health-related technology through trusted social relationships, and design support that aligns with how residents are actually learning.

## Introduction

As populations age worldwide, society must address the needs of older people in a manner that encourages a life that is dignified, purposeful, and enjoyable. This encompasses the creation of housing and community models tailored to this population, with just one example being the retirement village, also known as an Integrated Retirement Community (IRC). These are purpose-built communities where people (usually restricted to those over the age of 65) can live in their own individual homes, but with extra support from a variety of staff and services to support their well-being. These villages are becoming increasingly popular as they support independent living and can improve physical and psychological well-being for residents [1]. In the United Kingdom, this market is still small, with only 1% of those over 65 residing in an IRC [2], but is undergoing rapid expansion due to large investment [3]. At the same time, a plethora of assistive technologies is being proposed and developed to support older adults in maintaining their health and independence. Looking forward, retirement village providers are looking to support their residents to both understand and use existing technology for day-to-day tasks, and to foster innovation to move forward assistive technology.

This research was conducted alongside the technology and innovation department within a United Kingdom retirement village operator, with the aim to improve technology service provision for their residents. This particular later living housing provider was looking for appropriate and forward-thinking ways to support and enhance the lives of residents using technology, by better understanding technology use within their villages and examining uptake of their “Smart Market” scheme. The Smart Market was a scheme devised to encourage exploration and remove risk from trying new technologies by providing them free of charge to residents. They were then allowed to trial them for 6‐8 weeks, giving them an extended period to use and live with the device. After this, they could buy their own device if desired [4].

Research indicates that older people are open to learning and using new technology [5] but may be concerned about costs, privacy, and complexity [6-9]. Adoption amongst older people is influenced by usefulness (both perceived and actual), ease of use and learning, and the context in which the technology is used (including social, environmental, and personal contexts) [10]. Facilitating conditions can encourage adoption [11], including digital literacy education provision and a supportive environment under which people feel safe learning through trial and error [12,13]. The self-efficacy and confidence of each individual will also affect how they approach technology, as well as their cognitive and physical abilities [14]. While overall technology use is lower among older adults, substantial differences in technology attitudes persist within older age groups. These differences are patterned by gender and ethnicity [15,16], with individuals who identify as white and men reporting higher levels of technology use that are less pronounced in younger cohorts. Therefore, there are some clear guidelines on developing or providing suitable technology for older people, but some barriers still prevent true and equitable access that accommodates the full diversity of needs, including the personal, social, economic, and environmental.

Since the COVID-19 pandemic, many aspects of daily life have moved online, and research worldwide has indicated increased use of technology among older adults, particularly for staying connected and combating loneliness [17,18]. While many of the same barriers to using technology remain, more facilitators became apparent, including increased personal knowledge, help from friends and family, and social influences [19]. Accessing the internet regularly was perceived as more useful for socializing, but less useful for health care applications, compared to prepandemic levels [20]. Despite this increased technology use across many countries, older people without smartphone capabilities could be left isolated and dependent on relatives to be able to go about their daily lives [21]. Studies have shown an increased uptake of smart technologies in a variety of countries, but there are some indicators that adoption has varied by location and culture [22]. Yet, there is a persistent belief that many older adults are hesitant or unable to adopt new technology, even if it could improve their lives. A recent review by Mannheim points out the ageism in the discourse and practice of designing digital technology [23]; therefore, it is worth exploring how we understand the use of technology by older people and how service providers (and others who support older adults) can facilitate uptake.

This research examines how technology use among older adults living in retirement villages has evolved over recent years, with particular attention to the COVID-19 pandemic as an inflection point. By tracing shifts in everyday technology use, we aim to build a nuanced understanding of how digital tools are adopted and used by older adults. These insights are intended to inform the design and deployment of assistive technologies and services, particularly to support those enabling older adults to use technology in ways that promote independence and healthy aging.

## Methods

### Research Design

This repeated cross-sectional study included two questionnaires: one conducted in February and March 2020 (the mo immediately preceding the COVID-19 pandemic) and a follow-up conducted in June and July of 2023 (after pandemic restrictions had been lifted). Therefore, we conducted the surveys within the same retirement village setting, but not necessarily with the same participants. The timing of the initial survey was coincidental, but we identified an opportunity to readminister the questionnaire postpandemic to see if any changes had occurred within the retirement village population. Additional interviews were conducted with participants who took part in the Smart Market scheme, organized by the villages’ housing provider. A mixed methods approach was adopted because neither strand alone could fully address the aims of this study. The surveys captured broad patterns in technology use across a larger sample, while the interviews provided depth and context to help explain those patterns. The 2 strands were conducted sequentially, with interviews following the 2023 survey, and were integrated at the interpretation stage through a joint display shown in Table 1. The qualitative component is reported in accordance with the COREQ (Consolidated Criteria for Reporting Qualitative Research) checklist [24], and the overall mixed methods design follows the GRAMMS (Good Reporting of A Mixed Methods Study) guidelines [25].

**Table 1.** Joint display: integration of quantitative and qualitative findings.

Topic	Quantitative finding	Qualitative finding	Integration
Technology confidence	Significant increase in self-reported confidence from 2020 to 2023 (*M* 2.29 to 3.36; *P*<.001)	Interviewees described growing comfort through everyday use, fond recollections of past tech achievements suggested longstanding but previously unmeasured familiarity	Expansion: qualitative data suggest confidence increase reflects accumulated experience, not just pandemic-driven change
Gender gap in confidence	Men reported significantly higher confidence across all surveys (RV2023: *P*=.007, *r*=0.42)	Not directly addressed in interviews (sample too small to explore gender patterns)	Unresolved: quantitative finding not explained by qualitative data; identified as area for future research
Smart Market uptake	Low uptake: 17 of ~600 residents participated despite scheme removing cost and support barriers	Interviewees already owned multiple devices; those who participated found some devices inaccessible (especially wearables)	Explanation: qualitative data explain low uptake: the target population may have moved beyond introductory offerings, and accessibility barriers limited appeal
Limited use of features	High device ownership and internet use, but survey did not measure depth of feature engagement	No interviewees used health features (medication reminders, health tracking) despite being provided user guides	Expansion: qualitative data reveal a gap between ownership and meaningful use not measured in the survey
Pathways to learning	Self-taught and learning from friends or family increased significantly (*P*<.001)	Interviewees described helping neighbors, encouraging peers, and turning to friends before formal support	Confirmation: both strands confirm peer and informal learning as dominant pathways; interviews reveal the social and reciprocal nature of this support
Support needs and trust	Survey showed shift toward self-directed and informal learning; formal training declined	Interviewees valued trusted support but expressed anxiety about unknown providers and scams; village staff seen as reliable	Expansion: qualitative data add nuance: while formal training is declining, trusted and accessible support remains essential when things go wrong

### Context

This research was conducted within retirement villages across the United Kingdom from a single housing provider, where older people live in their own separate homes within a larger community. The housing provider has 20 sites, with more than 4200 residents in England. As well as providing communal spaces for those living within the community, villages will often include additional health and well-being services, fitness facilities, and social activities. The Smart Market is one example of these additional services, which provided the basis for this research. The lead researcher, a woman PhD student with previous experience in industry research and training in qualitative methods through her doctoral program, was undertaking this study as part of an internship with the housing provider’s technology and innovation department.

### Technology Adoption Survey

Both questionnaires contained two sections: demographics and current technology use. Participants were asked 6 demographic questions covering gender, age range, relationship status, ethnicity, education, and tenure within the village. Gender was assessed through self-identification. The 2023 survey response options (female, male, nonbinary, other, and prefer not to say) were designed to replicate those used in the 2020 survey. Although the response options used the terms female and male, the question assessed self-identified gender rather than sex assigned at birth, and results are therefore reported using the terms woman, man, nonbinary, other, and prefer not to say. Tenure type reflected the provider’s mix of housing arrangements, comprising leaseholders, shared owners, and social renters, a core feature of these particular communities.

The section on technology use contained 12 questions. First, participants were asked about their confidence with technology through the question: “How confident are you with technology such as smartphones, smart TVs, and tablets?” Responses were given on a 5-point Likert-type scale ranging from “not at all confident” to “very confident.” A follow-up multiple-choice question (with an open write-in option) asked how participants had gained this confidence. Here we define technology confidence as an individual’s self-assessed ability to use the common digital devices listed in the question. This was assessed using a single self-report item to maintain consistency with the housing provider’s existing surveys and to minimize respondent burden.

Subsequent questions covered broadband access, device ownership and use, and the types of devices used to connect to the internet. Participants were also asked which common online activities they engaged in and how frequently, their preferred method of contact, as well as how often they used the computer suite available within their village. Finally, they were asked whether they felt they understood what a smart device is, followed by whether they would be open to using one, and then given space to elaborate in their own words. The questionnaire was not pretested but was based on previous examples administered by the housing provider. The full questionnaire is available in Multimedia Appendix 1.

### Sample Characteristics

The sample comprised residents of retirement villages operated by a single service provider in England. All residents aged 55 years or older were eligible to participate; however, the average age of residents at the housing provider was 81 years, indicating that the majority of respondents would be much older. Recruitment followed a convenience sampling approach, reflecting both practical research constraints and an inclusive intention to allow any interested resident to take part. The only eligibility criterion was current residency within the village. To minimize duplicate entries, participants were asked to provide their apartment numbers as unique identifiers.

The sample was broadly representative of the retirement village populations in terms of age, gender, and ethnicity, aligning with national demographic patterns for adults older than 65 years in England [26]. Participants also represented a range of socioeconomic backgrounds. However, as only a small proportion of older adults in the United Kingdom reside in retirement villages, the sample may not fully reflect the wider community-dwelling older population.

### Survey Administration

The 2020 survey was conducted at 19 residential villages in England, with approximately 4000 residents, including the site at which the 2023 survey was to be conducted. The survey was administered both in person by door-to-door volunteers and through self-administration online (using Survey Monkey [27]), from February 21, 2020, to March 26, 2020, inclusive. It took approximately 15 minutes to complete.

The 2023 survey was only conducted at a single village, with approximately 400 residents, and was self-administered through paper copies. We distributed paper surveys to ensure that we reached people who did not use the internet, and due to limited resources, we were unable to go door-to-door at the village or visit multiple sites. The survey was advertised at a monthly village meeting, and people were asked to collect a survey pack from the foyer and return it to the same place upon completion. Surveys were available from June 29, 2023, to July 31, 2023, inclusive, before we collected and digitized the results. For paper-based responses, data were entered manually and then independently double-checked to minimize transcription errors.

Approximate response rates were 41% across all sites in 2020 (1707 responses from approximately 4200 residents), 54% at the retirement village site in 2020 (216 responses from approximately 400 residents), and 11% in 2023 (45 responses from approximately 400 residents). Exact rates cannot be calculated as village occupancy fluctuates. The lower response rate in 2023 likely reflects the change in administration method, from door-to-door distribution to self-collection from a communal area.

### Statistical Analysis

Results from the survey were compiled and statistically analyzed using Python and the SciPy Toolkit [28], looking for changes over time, as well as between different demographic groups within the retirement villages. We identified influencing factors that are known to affect technology use for comparison, including age group, gender, and socioeconomic status [15]. To categorize socioeconomic status, we used two possible signifiers: village tenure and the income deprivation affecting older people index (IDAOPI) [29], a measurement of the number of people aged over 60 within an area who experience income deprivation, to categorize the socioeconomic status of each village’s postcode. For village tenure, people living within the retirement villages are either leaseholders, shared owners, or social renters. Leaseholders are likely to be the wealthiest who own their properties, followed by shared owners, then social renters.

Analyses were conducted using nonparametric methods appropriate for ordinal and categorical data. Differences in Likert-type measures (such as technology confidence and computer suite use) were encoded on a 1‐5 scale and examined using Kruskal-Wallis *H* tests, with pairwise Mann-Whitney *U* comparisons and Benjamini-Hochberg (BH). Binary and multiple-response variables (eg, device ownership, internet access, online activities, and contact preferences) were analyzed using chi-squared tests of independence, also adjusted for multiple comparisons using the BH false-discovery-rate procedure. Effect sizes are reported as epsilon-squared (E²) for Kruskal-Wallis tests, rank-biserial r for Mann-Whitney tests, and Cramer *V* for chi-squared analyses. All tests used 2-tailed significance.

Questionnaires with substantial missing data (3 or more unanswered items) were excluded from analysis. Single missing answers were treated as item-level omissions and excluded from analyses of those specific measures. The overall rate of missing items was low (<5%) and appeared to occur at random, primarily due to occasional item nonresponse rather than systematic patterns; therefore, no imputation procedures were applied.

### Smart Market Interviews

The Smart Market interviews were conducted at 2 retirement village sites, where residents of both had participated in the 2020 survey. Available technology for trial is shown in Table 2.

**Table 2.** Technology available from the smart markets.

Technology	Devices	Recommended for…
Digital assistants	Amazon Echo Dot; Amazon Echo Show	Visual, hearing, and cognitive impairment, and arthritis
Fitness trackers	Fitbit Charge 2; Fitbit Inspire 2	Sleep and activity monitoring
Smart home	Philips Hue Bulbs; Amazon Smart Plug	Cognitive impairment and arthritis

### Recruitment

Participants who took part in the Smart Market trial and opted for home installation were asked if they would be willing to take part in an interview about their experience of using the technology. The Smart Markets were conducted at 2 of the previously surveyed villages, with approximately 600 residents combined. The Smart Market stall was set up in a high-traffic public area of each village, and the scheme was introduced to the village at a monthly meeting. The researcher invited passing residents to engage with the technology and made recommendations that would suit their wants or needs. If they opted in to the trial, they would then have the option to arrange installation with the researcher. As a result, the researcher had met all interview participants prior to the interview, having assisted with device installation in their homes. Participants were aware that the researcher was a PhD student undertaking an internship with the housing provider, and that both the researcher and provider were interested in understanding their patterns of technology use and how they could be further supported. Participants were also given a user guide, produced by the housing provider and tailored for older adults, which explained basic functionality and highlighted assistive health features, such as setting medication reminders. Those who opted in were asked to take part in an end-of-trial interview. In total, 17 people took a device from the Smart Market and 9 opted for installation. Of these, one was unable to take part in an interview due to time constraints, resulting in a convenience sample size of 8 participants.

### Interview Protocol

The initial trial period was 6‐8 weeks; however, due to factors including difficulty arranging collection dates and participant illness, the actual trial period was often longer, up to 3 months. Upon completion, the lead researcher collected the devices, and they were asked to participate in an interview to give feedback on the device, their day-to-day technology use, and technology support provision by the housing provider more generally. The interviews were semistructured and followed a topic guide developed by the research team in conjunction with the housing provider. These were pilot-tested with older people who did not live at a retirement village, which ensured clarity of language. Interviews were conducted one-on-one with the lead researcher at the participant’s residence. All interviews took under an hour, with the shortest duration 23 minutes, the longest 37 minutes, and the average 32 minutes. Supplementary notes were made by the researcher after each interview.

### Thematic Analysis

Audio recordings of the interviews were transcribed and analyzed thematically using NVivo (Lumivero) software. Following a qualitative descriptive approach, reflexive thematic analysis was used with a combined inductive and deductive strategy [30]. Initial themes were deduced from the interview structure (such as how older people currently use technology, and barriers and opportunities to adoption), and other codes and themes were identified during analysis. The lead researcher analyzed each interview 3 times and discussed results with the other researchers at regular meetings. Given the small sample size, we did not assume data saturation was reached, but qualitative interviews are intended to provide illustrative insights to complement survey findings.

### Ethical Considerations

The products available through the Smart Markets scheme were evaluated and approved for safe use by the housing provider. Participants in the 2020 survey received no compensation. In the 2023 survey, participants were entered into a prize draw for 1 of 5 £10 (US $13.50) shopping vouchers. Interviewees received no monetary compensation but were offered additional technology assistance from the researcher. All research was approved by the University of Nottingham ethics committee, reference number CS-2022-R54. Personal identifying information (eg, names and addresses) was not collected to minimize the risk of participant identification. Although complete anonymity cannot be guaranteed, all reasonable measures were taken to protect confidentiality. Data were stored on secure, password-protected servers.

## Results

### Survey

Henceforth, the 2023 survey is referred to as RV2023 (retirement village surveyed in 2023), the 2020 survey at the same site as RV2020 (retirement village surveyed in 2020), and all other 2020 sites combined as OTH2020 (other sites surveyed in 2020). This allows comparison of changes within the same village over time, as well as against the wider sample.

### Demographics

Participant demographics in the RV2023 survey were comparable to both the RV2020 and all other sites in OTH2020. Participant demographics are shown in Table 3.

**Table 3.** Comparison of survey demographics.

Characteristics	RV2020^a^, (N=216)	RV2023^b^, (N=45)	OTH2020^c^ (N=1491)
Gender, n (%)			
Woman	139 (64.4)	32 (71.1)	989 (66.3)
Man	77 (35.6)	12 (26.7)	502 (33.7)
Prefer not to say	—^d^	1 (2.2)	—
Age (years), n (%)			
Under 60	—	—	16 (1.1)
60‐69	15 (6.9)	2 (4.4)	162 (10.9)
70‐79	74 (34.3)	19 (42.2)	537 (36)
80‐89	99 (45.8)	18 (40)	607 (40.7)
Over 90	28 (13)	6 (13.3)	169 (11.3)
Ethnicity, n (%)			
White – British	206 (95.4)	44 (97.8)	1334 (89.5)
White – Irish	5 (2.3)	1 (2.2)	47 (3.2)
White – Other	4 (1.9)	—	23 (1.5)
Black – Caribbean	—	—	50 (3.4)
Other	1 (0.5)	—	37 (2.4)
Tenure, n (%)			
Leaseholder	47 (21.8)	24 (53.3)	545 (36.6)
Shared owner	115 (53.2)	15 (33.3)	462 (31)
Social renter	53 (24.5)	6 (13.3)	484 (32.4)
Temporary renter	1 (0.5)	—	—

a RV2020: retirement village surveyed in 2020.

b RV2023: retirement village surveyed in 2023.

c OTH2020: other sites surveyed in 2020.

d Not available.

Respondents to the survey, as is reflective of the villages themselves, were overwhelmingly White British. All participants were over 55, with the majority aged over 70 in all survey groups, although skewed slightly younger in RV2023, where more participants were aged 70‐79 years; this was not significant. More participants identified as women than men in each case. One major difference between RV2023 and the other surveys is that a majority in RV2023 reported that they were leaseholders, compared to a majority of shared owners in RV2020, and an almost even split for OTH2020 participants.

### Technology Attitudes and Adoption

Self-reported technology confidence differed across survey cohorts, as shown in Figure 1. Mean confidence was highest among RV2023 participants (mean 3.36, SD 1.18), compared with LH2020 (mean 2.29, SD 1.42) and OTH2020 (mean 2.40, SD 1.41). A Kruskal-Wallis test indicated a significant effect of survey cohort on technology confidence (*H(*2)=24.45; *P*<.001) with a small effect size (η²ₕ=.013). Post-hoc pairwise comparisons using Mann-Whitney *U* tests with Bonferroni correction showed that RV2023 participants reported significantly higher confidence than both LH2020 (*U*=2665; *P*<.001) and OTH2020 (*U*=45,737.5; *P*<.001). No significant difference was observed between LH2020 and OTH2020 (*U*=152,265.0; *P*=.52).

**Figure 1.** Technology confidence by survey. RV2020: retirement village surveyed in 2020; RV2023: retirement village surveyed in 2023; OTH2020: other sites surveyed in 2020.yy.OTH2020: Oother sites surveyed in 2020; RV2020: Rretirement village surveyed in 2020; RV2023: Rretirement village surveyed in 2023..; OTH2020: other sites surveyed in 2020.

An additional measure in RV2023 indicated that participants liked making use of technology. When asked to rate their enjoyment on a similar Likert-type scale (RV2023 mean 3.8, SD 1), indicating that they enjoy using technology “a little.”

Confidence with technology was developed through a wider range of means in 2023 compared with earlier cohorts. RV2023 participants were more likely to report being self-taught or having learned from friends or family than both 2020 groups. For example, 69% (31/45) of RV2023 participants reported learning from friends or family, compared with 20% (44/216)‐28% (416/1491) in 2020, and 53% (24/45) reported being self-taught, compared with 26% (57/216) in 2020. Fewer participants reported gaining confidence through optional training provided by the village (2/45, 4%) or in employment (9/45, 20%), with similar proportions across years. Chi-squared tests confirmed significant group differences for learning from friends or family (*χ*²_2_=43.54, *V*=.16; *P*<.001) and self-taught learning (*χ*²_2_=14.35, *V*=.10; *P*<.001), indicating small to medium effects. These results are shown in Figure 2.

**Figure 2.** How participants developed confidence with technology. RV2020: retirement village surveyed in 2020; RV2023: retirement village surveyed in 2023; OTH2020: other sites surveyed in 2020.yy.OTH2020: Oother sites surveyed in 2020; RV2020: Rretirement village surveyed in 2020; RV2023: Rretirement village surveyed in 2023).; OTH2020: other sites surveyed in 2020.

Participants in RV2023 were more likely to report that they understood what a smart device is (RV2023=69%, 31/45, RV2020=34%, 74/216, OTH2020=39%, 585/1491), and that they were open to using new smart technologies (RV2023=91%, 41/45, RV2020=39%, 85/216, OTH2020=40%, 589/1491) than participants in either 2020 group. This indicated significant associations between group and both understanding of smart devices (*χ*²_2_=46.33, *V*=.17; *P*<.001) and openness to new technology (*χ*²_2_=56.90, *V*=.18; *P*<.001), showing a medium effect size.

Smart technology ownership also increased substantially as shown in Figure 3. Across all devices, RV2023 participants reported higher use than both 2020 groups. For example, 91% (41/45) of RV2023 participants reported using a mobile phone, compared with 61% (131/216) of RV2020 and 64% (951/1491) of OTH2020 participants; similarly, 62% (28/45) of RV2023 participants reported using a tablet, compared with 33% (72/216)‐40% (596/1491) in the 2020 groups. Effect sizes were small to medium, indicating consistent but modest differences in device use across survey years. The largest differences were observed for mobile phones (*χ*²_2_=15.45, *V*=.09; *P*=.003), tablets (*χ*²_2_=13.24,* V*=.09; *P*=.004), and home computers (*χ*²_2_=10.92, *V*=.08; *P*=.008).

**Figure 3.** Smart device ownership. RV2020: retirement village surveyed in 2020; RV2023: retirement village surveyed in 2023; OTH2020: other sites surveyed in 2020.pp.OTH2020: Oother sites surveyed in 2020; RV2020: Rretirement village surveyed in 2020; RV2023: Rretirement village surveyed in 2023.; OTH2020: other sites surveyed in 2020.

Across all device types, RV2023 participants reported higher use of each device to connect to the internet than both 2020 groups. For instance, 69% (31/45) of RV2023 participants reported using a mobile phone to connect to the internet, compared with 33% (72/216) of RV2020 and 40% (600/1491) of OTH2020 participants. Similarly, 62% (28/45) of RV2023 participants reported using a tablet, compared with 33% (72/216)‐36% (542/1491) in the 2020 groups, and 62% (28/45) reported using a home computer, compared with 33% (72/216)‐38% (573/1491) previously. Effect sizes were small to medium. The largest group differences were observed for mobile phones (*χ*²_2_=19.65, *V*=.11; *P*<.001), tablets (*χ*²_2_=16.69, *V*=.10; *P*<.001), and home computers (*χ*²_2_=13.14, *V*=.09; *P*=.003).

The number of people with a home internet connection also showed a notable increase. RV2023 participants were substantially more likely to have an internet connection (40/45, 89%) compared with RV2020 (108/216, 50%) and OTH2020 (975/1491, 65%) respondents. Access to an internet connection differed significantly between groups (*χ*²_2_=31.76, *V*=.14; *P*<.001). The effect size indicated a small to medium association between survey group and internet connectivity, suggesting a marked increase in internet access.

Use of the internet also differed significantly between groups. Across all activities, RV2023 participants reported higher engagement than both 2020 groups. For example, 80% (26/45) of RV2023 participants used the internet to find information, compared with 50% (108/216)‐52% (771/1491) in 2020, and 76% (34/45) reported using it to keep in touch, compared with 44% (96/45)‐46% (686/1491) previously. Similarly, 64% (29/45) reported using the internet for online banking and online shopping, compared with 27% (59/216)‐31% (462/1491) in the 2020 groups. Chi-squared tests indicated significant group differences for online banking (*χ*²_2_=25.51, *V*=.12; *P*<.001), online shopping (*χ*²_2_=19.95, *V*=.11; *P*<.001), keeping in touch (*χ*²_2_=15.77, *V*=.10; *P*<.001), and finding information (*χ*²_2_=14.50, *V*=.09; *P*=.001). Effect sizes were small to approaching medium, indicating consistent increases in everyday internet use across survey years.

Preferred contact methods also differed significantly between groups. RV2023 participants were more likely to prefer email communication than either 2020 group, with 73% (33/45) selecting email compared with 35% (75/216 and 521/1491) in both 2020 surveys. Phone contact remained the most common overall, reported by 82% (37/45) of RV2023 participants, 89% (192/216) of RV2020, and 78% (1159/1491) of OTH2020 participants. Few participants in any group selected social media (<4%). Significant differences were found for email (*χ*²_2_=28.12, *V*=.13; *P*<.001) and phone contact (*χ*²_2_=14.52, *V*=.09; *P*<.001).

Frequency of computer suite use differed significantly between groups (*H*(2) = 8.37, *E*²=.004; *P*=.02). However, mean usage was low across all groups, with RV2023 participants reporting slightly higher mean use (mean 1.42, SD 0.79) than RV2020 (mean 1.33, SD 0.74) and OTH2020 (mean 1.28, SD 0.83). Effect sizes were small (*r*=0.03-0.06), indicating minimal practical differences in computer suite use between groups.

### Factors Affecting Technology Adoption

No significant difference was found between villages based on socioeconomic factors when comparing them by the IDAOPI of the local area. However, when examining device ownership by tenure, disparities emerged, particularly in earlier surveys. In OTH2020, leaseholders reported higher ownership of home computers (282/545, 52%), tablets (258/545, 47%), and Kindles (109/545, 20%) compared with shared owners and social renters (*χ*²_2_=53.53‐19.36, *V*=.11–.19; *P*<.001). A similar pattern was seen in RV2020, where ownership of home computers (*χ*²_2_=9.11, *V*=.21; *P*=.05) and Kindles (*χ*²_2_=9.54, *V*=.21; *P*=.05) was significantly higher among leaseholders. Differences by tenure had largely equalized by RV2023, with no significant differences in ownership across device types. Leaseholders and shared owners reported broadly similar access to most technologies, while social renters showed slightly higher ownership of mobile phones (6/6, 100%), digital assistants (2/6, 33%), and tablets (5/6, 83%).

Across all surveys, men reported higher technology confidence than women. For example, in RV2023, men were more confident than women (Men: mean 4.17, SD 0.94, Women: mean 3.06, SD 1.13, *r*=0.42; *P*=.007). In RV2020, men exceeded women (men: mean 2.61, SD 1.46, Women: mean 2.09, SD 1.38, *r*=0.19; *P*=.007). In OTH2020, men also exceeded women (Men: mean 2.57, SD 1.48, Women: mean 2.29, SD 1.39, *r*=0.09; *P*=.002).

Effect sizes ranged from small to moderate, largest in RV2023. Age-related differences were evident in 2020 but not in 2023. In RV2020, confidence decreased with age (60‐69: *M*=3.43, 70‐79: *M*=2.58, 80‐89: *M*=2.14, 90+: *M*=1.36), *H*(3) = 30.07, *E*²=.13; *P*<.001). In OTH2020, the same pattern held (60‐69: *M*=3.13, 70‐79: *M*=2.83, 80‐89: *M*=2.01, 90+: *M*=1.49), *H*(3) = 224.24, *E*²=.15; *P*<.001). In RV2023, age bands did not differ significantly (*H*(3) = 1.74; *P*=.63).

### Interviews

From 17 people who took part in the Smart Market trial, 8 opted to participate in an interview. The following section includes the results of thematic analysis of these interviews, and information about participants can be seen in Table 4. We began with predefined themes, developing codes based on discussion of participants’ experience with the Smart Markets and their chosen technology, with results presented in the “Smart Markets Experience” subsection. However, we also sought to identify how people used other technology in their everyday lives. This more open-ended conversation is discussed under the theme of “Other Experiences with Technology,” including themes related to how technology is used in the participants’ past and present, and how they could be helped in the future.

**Table 4.** Interview participant information.

ID	Gender	Age group (years)	Device trialed	Device purchased?
A01	Woman	75‐79	Fitbit Inspire 2	No
A02	Man	75‐79	Fitbit Charge 2	No
A03	Woman	70‐74	Echo Dot	Yes
A04	Woman	75‐79	Echo Show	Yes
B01	Man	85‐89	Echo Dot	No
B02	Man	80‐84	Smart plug	Yes
B03	Man	60‐64	Philips Hue Lights	Yes
B04	Woman	75‐79	Fitbit & Echo Dot	No and yes

### Smart Markets Experience: Fitness Trackers

Response to Fitbits was overwhelmingly negative from our 3 interview participants, despite the fact that the device was one of the most popular to trial. Participants found it difficult to use, often due to conditions which are common among older people; for example, participants with reduced vision could not see the small screen, and those with reduced dexterity could not operate it. Overall, participants found it “too fiddly” to use and difficult to charge. Additionally, participants also felt the device was uncomfortable and irritating on the skin. Participants also found the app too complex to use.

Those trialing a Fitbit had hoped to be able to better monitor their well-being and help themselves better manage health decisions, and some had even had the Fitbit recommended by a doctor. They often expressed disappointment and frustration that they had not found it useful, with one participant saying:

I felt it was going to do something for me. Disappointed when it didn’t. I liked the look of it, I liked the idea of it, and I think if I could get one that could be more specific it would be very, very useful and something you’d keep up with.

[LH02]

### Digital Assistants

In contrast, the 4 participants trialing Echo Alexa devices found them enjoyable and easy to use, and 3 out of 4 bought the device at the end of the trial, with the fourth stating he was likely to buy one in the future. Participants all stated that it took them only a matter of minutes to learn how to use the device, and that its operation was highly intuitive. Echo products were used primarily for entertainment, with many using them to listen to their favorite music and radio stations, or for viewing their favorite photos. One participant said that having easy access to music had helped her, stating:

When I got ill, I lost my interest in music. So it’s helped me a bit like that. It’s helped my mental health a little.

[LH03]

Most participants also used the devices for information finding, shopping lists, and setting reminders. However, despite providing the participants with a user guide during installation, none used the devices for health and well-being purposes or made use of anything like the full range of functionality supported by Alexa.

### Smart Home Devices

The single participant who opted to loan a smart plug had also found it intuitive and easy to use, and had mainly used it for entertainment purposes (switching off and on his television), but had also tried it with a lamp and fan. Similarly, the participant using the smart bulbs largely used them for dimming the lights while watching films, but also set them up to an evening timer. He also enjoyed exploring and learning how to use the devices. Both participants already owned Echo products and were familiar with using Alexa. Both opted to buy their own devices at the end of the trial period.

### Other Experiences With Technology: Using Technology Today

All of our participants were relatively comfortable using technology, and often owned smartphones, smart TVs, and tablets. They used this technology for entertainment, information finding, reminiscing, keeping in touch, accessing online services, and online shopping. Participants were comfortable using online services and used them frequently; however, many were concerned about services, particularly banks, moving toward “online only.”

The 6 participants who used video calling to speak with relatives in other parts of the country, or sometimes in other countries, valued this highly. They spoke of the happiness they felt when they were able to see as well as hear their loved ones and would often encourage other friends within the village to engage with the technology.

They also often used technology to help monitor or manage impairments; for example, 1 participant used a blood glucose monitor connected to his smartphone. Others with hearing impairments used hearing aids which connected to their other devices, or specialist in-ear headphones to watch the TV. Another, who has some limitations after experiencing a stroke, has a low level of home automation set up to negate mobility and dexterity issues. Finally, a participant with visual impairments uses large format clocks, and text-to-speech on his mobile phone.

### Past Experiences With Technology

Our participants often had fond stories to tell about their past experiences with technology, either used at work, or for projects and hobbies. They were often early workplace adopters, and 2 participants reported that they were the first people to be trained on a computer at their office when they were first introduced. Some had proud achievements which they linked with technology, including app development, computer-aided design, and documentary making. Realizing important objectives in their lives with the aid of technology was often regarded with a sense of joy and accomplishment.

### Helping and Being Helped With Technology

Despite relatively high levels of technological literacy among our participants, they had all, at some point, needed help from others. Sometimes they received help from staff at the residential village, and other times from paid external computer repair companies. However, they sometimes felt it difficult to trust unknown people with devices containing personal information, especially as many expressed concerns about scammers using technology. In other, less critical, circumstances they would turn to friends or neighbors for help. In turn, they were also keen to help others when they had knowledge about a problem or piece of technology and often encouraged their neighbors to use technology more in their lives.

### Integration of Results

To illustrate how the quantitative and qualitative strands were integrated, Table 1 presents a joint display summarizing the main findings [31]. Each finding is categorized as confirmation (both strands converge), expansion (qualitative adds depth beyond the survey data), explanation (qualitative contextualizes a quantitative pattern), or unresolved (qualitative data could not address the quantitative finding). Combining methods often revealed insights that neither the survey nor interviews would have produced alone, and our integrated findings are discussed in the subsequent section.

## Discussion

### Principal Findings

Drawing on the integrated findings presented in Table 1, the following sections discuss the key themes emerging from this study. These include shifting patterns of confidence and adoption, the role of informal and peer-based learning, the persistent gender gap, and implications for retirement village technology provision.

### Shifting Patterns of Technology Use, Confidence, and Adoption

Survey results show a clear increase in technology use and adoption within older people in these retirement villages across several measures, including device ownership, what they use the internet for, and, perhaps most importantly, confidence in technology use. It also seems apparent that confidence in using technology has grown rapidly among this cohort since the beginning of the COVID-19 pandemic.

Similar patterns of pandemic-accelerated technology adoption among older adults have been observed in Canada [20], Saudi Arabia [21], Spain [17], and across a cross-cultural study of Spain, France, and Israel, which highlighted that knowledge, affordability, social support, and cultural context all shaped technology usability [22]. In the United States, the share of older adults using technology has grown steadily [32], with device ownership and prior experience identified as stronger predictors of engagement than age [33], and facilitators such as perceived usefulness and trusted support remaining important [34]. However, analysis of US national survey data from around the pandemic found that age-based disparities in digital health technology use actually widened in relation to health care, calling for better provision to ensure older people are not excluded from access [35].

We observed that in 2020, older participants felt less comfortable with technology, and it has been historically observed that as age increases, technology use decreases [36,37]. However, these differences were not present in the 2023 survey, suggesting that age may now play a reduced role in technology adoption compared with previous years, consistent with findings from studies of telehealth engagement showing that device access and prior experience are stronger predictors of use [33]. This shift may reflect generational change, as individuals now entering retirement are more likely to have engaged with digital technologies throughout their working lives, although this contrasts with previous findings suggesting that retirement may be associated with declining technology skills over time [38,39]. In comparing the demographic profiles of our 2 surveys, the 2023 sample skewed slightly younger. While the 3-year gap between surveys is insufficient for a complete generational shift, even new, younger arrivals at the retirement village could introduce individuals with greater technological confidence. However, differences in age distributions were not statistically significant and the average age within the villages remained around 81 years. These findings highlight the need to view technology engagement among older adults as dynamic and context-dependent, rather than primarily age-driven.

Between 2020 and 2023, smart devices continued to become more affordable and intuitive, and it is likely that improvements in usability and usefulness have contributed to this shift in confidence. At a local level, the retirement village provides resources that could support technology adoption, including free Wi-Fi in communal areas, a computer suite, and the Smart Market scheme described in this study, though uptake of these offerings was low. Computer suite use was minimal across all survey groups. Only 4% (2/45) of RV2023 participants reported gaining confidence through village-based training, though it should be noted that such training is optional and requires residents to actively sign up rather than being offered by default. Research has consistently identified socioeconomic factors such as income and education as significant barriers to technology adoption among older adults [9,37], yet in our study no significant differences in technology confidence were found based on indicators of socioeconomic status such as tenure type or IDAOPI. This may reflect the range of incidental technology exposure within retirement village communities, where residents encounter technology in use across everyday interactions, for example among peers, with staff, and through service provision, potentially building confidence through informal exposure rather than structured training. Overall, this suggests that the observed changes in confidence reflect a combination of societal, technological, and community-level factors, accelerated by the necessities of the pandemic.

We observed that the gender gap in technology confidence persisted across our surveys [15], with men reporting significantly higher confidence than women. Prior to the pandemic, being a man and having higher education were significant predictors of internet access [40], and subsequent research found that the gap in internet use narrowed after the pandemic, particularly for socializing and entertainment [41]. However, a systematic review of the gender digital divide worldwide found that second-level divides in confidence and meaningful use persist, with socioeconomic factors accumulated across the life course identified as a primary explanation [42]. These life-course factors are well documented among older British women. Women in older cohorts were more likely to follow trajectories characterized by lower educational attainment, career breaks for caring responsibilities, and concentration in lower-status occupations [43]. These cumulative patterns would have reduced their exposure to workplace computerization during the critical adoption period of the late 20th century. Notably, our women interview participants did describe encountering technology in the workplace, though not through high-status technical roles but through incidental exposure, such as being selected for training on computerized till systems while working on a shop floor. This suggests that even informal workplace exposure may be sufficient to build lasting confidence with technology. However, the women who took part in our interviews had self-selected into a technology trial, and are therefore likely to represent those with above-average interest in technology. The survey-level gender gap may better reflect the experiences of women who lacked even these incidental opportunities. As successive cohorts of retirees will have had greater and more equal exposure to workplace technology, future research should investigate whether this confidence gap diminishes over time or persists through other socioeconomic mechanisms.

### From Access to Meaningful Use: Barriers, Support, and Usability

Overall, 2023 survey respondents expressed a willingness to try new smart devices, aligning with the aims of the Smart Market scheme. However, Smart Market uptake was low, with only 17 of over 600 residents taking part, despite removing financial and support barriers. This may be because older adults now tend to either own or have experience of these smart devices, making the demand for these types of introductory offerings low. This is further supported by the fact that respondents from all surveys did not make use of the computer suite provided at their village, as the majority have broadband connectivity and smartphones in their homes. Interviews found that participants commonly used digital devices for basic communication and information access, but rarely engaged with features such as medication reminders, appointment management, health tracking, accessibility settings, and smart-home controls, thereby limiting their assistive and health care potential. This aligns with previous studies identifying limited awareness of device functionality as a major barrier to effective use [34], and with evidence suggesting that older adults increasingly prefer to learn technology independently rather than through formal instruction manuals [44]. Together, these findings point to the need for alternative approaches that actively encourage discovery beyond traditional user guides.

This is not to imply that older adults no longer require support in accessing and using technology. Our interviewees repeatedly referenced a lack of support when something goes wrong and an uncertainty about support from outside sources. While technology support falls outside the scope of independent living provision, staff occasionally assist on an informal basis. While our data shows that older adults are more comfortable using their devices for day-to-day activities (such as shopping and banking), they can still struggle when technology malfunctions. It is important that they have access to reliable and trusted support when problems occur, especially given the high prevalence of scams targeting older adults and the potential negative impacts on well-being [45].

Additionally, interviewees expressed frustration with the inaccessibility of some devices available through the Smart Market scheme, particularly wearable devices that were not designed with common impairments in mind, which negatively affected usability and were likely to limit adoption [46]. As older adults are not the target market for consumer fitness devices, greater attention to their needs in device design may improve uptake [47]. Conversely, participants spoke enthusiastically about devices they already used to support specific impairments, including visual and hearing impairments, diabetes management, and stroke-related limitations. Taken together with their limited engagement with the more advanced features of other smart technologies, this suggests that clearer demonstrations of personal relevance and benefit could increase meaningful adoption. Overall, these findings highlight the need to facilitate access to innovations tailored to individuals’ specific abilities and needs, promoting necessary confidence and a sense of agency in personalized technology use [48].

### Self-Efficacy, Peer Learning, and Community-Based Support

A key finding from our survey is that increasing numbers of older adults are gaining confidence with technology through self-directed learning, consistent with prior studies showing a shift from reliance on user manuals toward independent trial-and-error approaches among more recent cohorts [44]. This may indicate increased self-efficacy and show that they are more confident and able to learn by themselves. The Smart Market scheme attempts to create an environment allowing for this type of experimentation, and interviewees felt they benefited from the support provided and the opportunity to explore technology at their own pace and in their own environment.

The survey indicated that increasing numbers of participants were learning to use technology through friends and family, encompassing both external family members and peer support. Interviews clarified that participants both received help from children and grandchildren outside the village, as well as supporting neighbors within the community who were struggling. While peer support and a strong sense of community are key strengths of retirement village living, overreliance on informal help alone risks placing undue burden on volunteer residents [49]. This suggests the value of hybrid support models that combine peer-based support with accessible, formal sources of technical education. Leveraging the growing confidence and skills of older adults in this way may help extend support to those who remain less comfortable with technology, while ensuring that responsibility does not fall disproportionately on a small number of individuals.

Positive technology experiences described by interviewees were frequently social in nature, including video calling family, using technology for hobbies, and helping neighbors learn. This pattern is consistent with wider evidence that social factors play a crucial but often overlooked role in facilitating technology adoption among older adults [9]. It suggests that the design of health-related technology interventions should incorporate a social dimension, for example, by embedding technology tips within existing social activities such as health and well-being groups, rather than expecting older adults to adopt them as individual self-management tools.

### Implications for Retirement Village Technology Provision

Despite the increase in use of technology and online services, including online banking and shopping, there are still a notable number of older people both within the retirement villages (around 11%, 5/45) and in the United Kingdom more broadly (around 18%) who do not have home internet access, with those who have not yet gotten online citing a lack of interest and cost as barriers to adoption [50]. Our interviewees, despite mostly using online banking themselves, also raised concerns about being unable to access in-person services when they felt the need. Therefore, while increasing technology confidence among older adults is an encouraging trend, support needs remain. A tension exists within the independent living model as retirement villages can foster engagement with technology, but the emphasis on resident independence places limits on the formal support they can reasonably offer.

While our results are reflective of the trends observed elsewhere in the United Kingdom [15], it is still worth reflecting on the microcosm of the retirement village, and what living in this community may mean. For example, we found no differences based on the deprivation index of the retirement village locality, which would be expected to have an influence on purchasing power [33]. Type of tenure at the retirement village also had a conflicting effect on levels of device ownership, with the recent survey indicating social renters owned more smart devices. Most of our survey participants identified as White British, which is reflective of the current over 65 population in the United Kingdom, but these trends should also be analyzed in older people from other ethnicities to ensure there is not a widening of the “digital divide” between people from different cultural backgrounds [16]. Furthermore, retirement villages offer older adults a prebuilt community and additional support, with the intention to improve residents’ well-being [1]. As this model becomes increasingly common, it is essential that providers remain responsive to technological developments and integrate them thoughtfully into the care and support of their residents, while minimizing negative effects such as privacy and agency violations [51,52]. However, such support must remain fluid and adaptive, recognizing both the rapid pace of technological change and the evolving capabilities, interests, and confidence of older adults demonstrated by our findings.

Future plans from the provider include conducting a long-term month smart properties evaluation with residents who are open to the idea of smart technology. There are also plans to invest in digital assistants for reception areas and well-being hubs in all locations, providing basic information such as activity planners to help residents familiarize themselves with the technology and reduce routine inquiries to reception staff. In addition, technology could be used to stream events virtually (such as village meetings) to help residents who are unable to attend in person stay connected and build confidence to participate in future events. Finally, there are plans to explore a dedicated care and smart technology project to encourage engagement from residents requiring greater assistance.

Altogether, we present an emerging technological literacy among older adults, through which housing and service providers can engage with their users to deploy technology. Older adults do not necessarily require basic support and access, but a more sustained and deep engagement with technology and its capabilities. By designing appropriate infrastructure and providing support, providers could improve well-being and independence among their residents. For example, video calling is already used by many and could help older adults stay in touch with friends and family, potentially reducing loneliness. Digital assistants, which are intuitive to use through voice control, could provide reminders for medication, entertainment to improve mental health, and be linked with home automation to provide physical assistance. Wearable devices could be used to allow residents and health care staff to monitor important health metrics. These advances can support older adults in maintaining independence, while also enabling staff to streamline their work and gather valuable data on residents to enhance efficiency and reduce costs.

However, integrating this technology within retirement village settings will require substantial upskilling and ongoing support across the workforce, efforts which are currently underway through the provider [53]. Roles in this sector are already physically and emotionally demanding and often undervalued in terms of pay. Staff must not only be equipped to support residents in using technology but also enabled to use these systems to make their work both more efficient and rewarding. Technology should therefore be designed and implemented to ease workloads, not to add additional burdens, undermine wages, or make this human support obsolete. Although our research does not include the perspectives of village staff, our findings indicate that human involvement remains central to technology set-up and sustained use, highlighting the importance of explicitly incorporating staff needs and expertise into future planning. At the same time, we observed emerging forms of peer support, whereby more confident residents assisted friends and neighbors, suggesting opportunities for complementary, community-based models of digital support that could help distribute effort while fostering social connection and mutual learning.

In summary, our findings point to several strategies for retirement village providers. The dominance of peer and informal learning suggests that providers could formalize these pathways, for example through digital champions or partnership schemes, that pair confident residents with those less confident. The underuse of health-related features indicates that simply providing the technology and relevant information is insufficient. This is notable given the potential of such features to support independent living and health self-management, functions particularly relevant to an older adult population managing, for whom timely reminders and health monitoring could reduce reliance on formal care. Instead, they should be actively introduced through trusted one-to-one interactions, drawing on social relationships that participants indicated as important to engagement. Retirement villages often already host regular health and well-being groups where residents meet to share experiences; these could provide a natural setting for introducing and discussing health-related technology features, such as medication reminders or activity tracking, in a way that is social rather than instructional. However, the persistent gender gap is unlikely to be addressed through training schemes alone, as it can be reflective of cumulative life-course disadvantages. Evidence that older women’s technology engagement increases when it becomes personally meaningful, such as the narrowing of the gender gap in internet use for socializing and entertainment [41], suggests that providers should focus less on generic digital skills programs and more toward embedded, socially oriented approaches that align with residents’ desires. Above all, our research suggests that technology provision for older adults is most effective when it becomes part of the social fabric of the community, rather than delivered as a standalone service.

### Limitations

This study compared 2 independent cross-sectional surveys rather than tracking individual participants. Due to anonymization, it was not possible to confirm whether the same individuals responded to the 2020 and 2023 surveys, limiting interpretation of changes over time. As such, observed differences between 2020 and 2023 cannot be interpreted as individual-level change and causal inferences should be drawn with caution.

Interview participants had opted into home installation of digital technologies, introducing a potential selection bias toward residents who are more engaged with or positive about technology use. Furthermore, the small interview sample means thematic saturation cannot be claimed. As a result, the qualitative strand was unable to explore all patterns identified in the survey data, most notably, the persistent gender gap in technology confidence. This is identified as a potential area for future research.

In addition, the sample lacked ethnic diversity, which may limit generalizability to more diverse populations. Finally, the researcher’s involvement in the wider context of technology provision and support may have influenced participants’ responses.

### Conclusions

Capturing a unique snapshot in time, surveys on technology use were conducted at retirement villages across England just prior to the 2020 COVID-19 lockdowns and then repeated at one village in 2023. In 2023, surveyed participants owned more technology, felt more confident using it, used it for more purposes, and indicated more openness to using smart devices in the future. Interviews with participants in a technology trial scheme provided complementary depth, revealing that positive experiences with technology were frequently social in nature.

Contrary to stereotypes, older adults in this setting are already using technology at significantly higher rates than in previous years, and age-related differences in confidence observed in 2020 had disappeared by 2023. A persistent gender gap remains; however, one that targeted training alone is unlikely to resolve given the cumulative life-course disadvantages discussed above. Broader shifts in confidence reflect the interplay of societal, technological, and community-level changes, with the pandemic serving as an accelerating rather than originating force.

Our findings point toward technology provision that is embedded in the social life of retirement communities rather than delivered as a standalone service. Practically, this means formalizing peer support pathways, introducing health-related features through trusted relationships, and connecting technology to the activities residents already value. Realizing this potential will require ongoing investigation into how technology can be integrated into daily life in these settings, and sustained attention to supporting the workforce that maintains these communities.

## Supplementary material

10.2196/92816 Multimedia Appendix 1 Questionnaire administered in 2020 and 2023.

## Abbreviations

BH: Benjamini-Hochberg
COREQ: Consolidated Criteria for Reporting Qualitative Research
GRAMMS: Good Reporting of a Mixed Methods Study
IDAOPI: Income Deprivation Affecting Older People Index
IRC: Integrated Retirement Community
OTH2020: other sites surveyed in 2020
RV2020: retirement village surveyed in 2020
RV2023: retirement village surveyed in 2023

## References

[1] Holland C, Boukouvalas A, Wallis S (2017). Transition from community dwelling to retirement village in older adults: cognitive functioning and psychological health outcomes. Ageing Soc.

[2] Bowles L, Wellman P, Donahue C (2022). Spotlight: UK senior living — the inflection point. https://pdf.euro.savills.co.uk/uk/spotlight-on/uk-senior-living---the-inflection-point.pdf.

[3] (2025). UK seniors housing market update. Knight Frank.

[4] Heron L (2023). Smart homes offer solutions for seniors ageing in place. The Institution of Engineering and Technology.

[5] Kuerbis A, Mulliken A, Muench F, A. Moore A, Gardner D (2017). Older adults and mobile technology: factors that enhance and inhibit utilization in the context of behavioral health. Ment Health Addict Res.

[6] Peek STM, Wouters EJM, van Hoof J, Luijkx KG, Boeije HR, Vrijhoef HJM (2014). Factors influencing acceptance of technology for aging in place: a systematic review. Int J Med Inform.

[7] Mitzner TL, Boron JB, Fausset CB (2010). Older adults talk technology: technology usage and attitudes. Comput Human Behav.

[8] Vaportzis E, Clausen MG, Gow AJ (2017). Older adults perceptions of technology and barriers to interacting with tablet computers: a focus group study. Front Psychol.

[9] Bertolazzi A, Quaglia V, Bongelli R (2024). Barriers and facilitators to health technology adoption by older adults with chronic diseases: an integrative systematic review. BMC Public Health.

[10] Ma Q, Chan AHS, Teh PL (2021). Insights into older adults’ technology acceptance through meta-analysis. Int J Hum Comput Interact.

[11] Peek STM, Luijkx KG, Rijnaard MD (2016). Older adults’ reasons for using technology while aging in place. Gerontology.

[12] Barnard Y, Bradley MD, Hodgson F, Lloyd AD (2013). Learning to use new technologies by older adults: perceived difficulties, experimentation behaviour and usability. Comput Human Behav.

[13] Mitzner TL, Savla J, Boot WR (2019). Technology adoption by older adults: findings from the PRISM trial. Gerontologist.

[14] Mostaghel R, Oghazi P (2017). Elderly and technology tools: a fuzzyset qualitative comparative analysis. Qual Quant.

[15] Prescott C (2019). Internet users, UK: 2019. Office of National Statistics.

[16] Mitchell UA, Chebli PG, Ruggiero L, Muramatsu N (2019). The digital divide in health-related technology use: the significance of race/ethnicity. Gerontologist.

[17] Dura-Perez E, Goodman-Casanova JM, Vega-Nuñez A (2022). The Impact of COVID-19 confinement on cognition and mental health and technology use among socially vulnerable older people: retrospective cohort study. J Med Internet Res.

[18] Murciano-Hueso A, Martín-García AV, Cardoso AP (2022). Technology and quality of life of older people in times of COVID: a qualitative study on their changed digital profile. Int J Environ Res Public Health.

[19] Haase KR, Cosco T, Kervin L, Riadi I, O’Connell ME (2021). Older adults’ experiences with using technology for socialization during the COVID-19 pandemic: cross-sectional survey study. JMIR Aging.

[20] Sixsmith A, Horst BR, Simeonov D, Mihailidis A (2022). Older people’s use of digital technology during the COVID-19 pandemic. Bull Sci Technol Soc.

[21] Alharbi RA, Altayyari FT, Alamri FS, Alharthi SA (2021). Pandemic-driven technology during COVID-19: experiences of older adults.

[22] Elimelech OC, Ferrante S, Josman N (2022). Technology use characteristics among older adults during the COVID-19 pandemic: a cross-cultural survey. Technol Soc.

[23] Mannheim I, Wouters EJM, Köttl H, van Boekel LC, Brankaert R, van Zaalen Y, Heyn PC (2023). Ageism in the discourse and practice of designing digital technology for older persons: a scoping review. Gerontologist.

[24] Booth A A, Hannes K, Harden A, Noyes J, Harris J, Tong A, Moher D, Altman DG, Schulz KF, Simera I, Wager E (2014). Guidelines for Reporting Health Research: A User’s Manual.

[25] O’Cathain A, Murphy E, Nicholl J (2008). The quality of mixed methods studies in health services research. J Health Serv Res Policy.

[26] Storey A (2023). Profile of the older population living in england and wales in 2021 and changes since 2011. https://www.ons.gov.uk/peoplepopulationandcommunity/birthsdeathsandmarriages/ageing/articles/profileoftheolderpopulationlivinginenglandandwalesin2021andchangessince2011/2023-04-03.

[27] Turn curiosity into clarity. Survey Monkey.

[28] Virtanen P, Gommers R, Oliphant TE (2020). SciPy 1.0: fundamental algorithms for scientific computing in Python. Nat Methods.

[29] (2019). Income deprivation affecting older people index (IDAOPI). Ministry of Housing, Communities and Local Government (MHCLG).

[30] Braun V, Clarke V APA Handbook of Research Methods in Psychology, Vol 2: Research Designs: Quantitative, Qualitative, Neuropsychological, and Biological.

[31] Guetterman TC, Fetters MD, Creswell JW (2015). Integrating quantitative and qualitative results in health science mixed methods research through joint displays. Ann Fam Med.

[32] Faverio M (2022). Share of those 65 and older who are tech users has grown in the past decade. https://pameladwilson.com/wp-content/uploads/Tusers-65-and-older_-Pew-Research-Center.pdf.

[33] Choi NG, DiNitto DM, Marti CN, Choi BY (2022). Telehealth use among older adults during COVID-19: associations with sociodemographic and health characteristics, technology device ownership, and technology learning. J Appl Gerontol.

[34] Harris MT, Blocker KA, Rogers WA (2022). Older adults and smart technology: facilitators and barriers to use. Front Comput Sci.

[35] Qiu Y, Huang H, Gai J, De Leo G (2024). The effects of the COVID-19 pandemic on age-based disparities in digital health technology use: secondary analysis of the 2017-2022 Health Information National Trends Survey. J Med Internet Res.

[36] Morris MG, Venkatesh V (2000). Age differences in technology adoption decisions: implications for a changing work force. Pers Psychol.

[37] Charness N, Boot WR (2022). A grand challenge for psychology: reducing the age-related digital divide. Curr Dir Psychol Sci.

[38] Cavapozzi D, Dal Bianco C (2022). Does retirement reduce familiarity with Iinformation and communication technology?. Rev Econ Household.

[39] Schuster AM, Cotten SR, Xie B (2024). Differences between employed and retired older adults in information and communication technology use and attitudes. Work Aging Retire.

[40] Huxhold O, Hees E, Webster NJ (2020). Towards bridging the grey digital divide: changes in internet access and its predictors from 2002 to 2014 in Germany. Eur J Ageing.

[41] Bünning M, Schlomann A, Memmer N, Tesch-Römer C, Wahl HW (2023). Digital gender gap in the second half of life is declining: changes in gendered internet use between 2014 and 2021 in Germany. J Gerontol B Psychol Sci Soc Sci.

[42] Acilar A, Sæbø Ø (2023). Towards understanding the gender digital divide: a systematic literature review. Glob Knowl Mem Commun.

[43] McMunn A, Lacey R, Worts D (2015). De-standardization and gender convergence in work–family life courses in Great Britain: a multi-channel sequence analysis. Adv Life Course Res.

[44] Pang C, Collin Wang Z, McGrenere J, Leung R, Dai J, Moffatt K (2021). Technology adoption and learning preferences for older adults: evolving perceptions, ongoing challenges, and emerging design opportunities.

[45] Kemp S, Erades Pérez N (2023). Consumer fraud against older adults in digital society: examining victimization and Its impact. Int J Environ Res Public Health.

[46] Moore K, O’Shea E, Kenny L (2021). Older adults’ experiences with using wearable devices: qualitative systematic review and meta-synthesis. JMIR Mhealth Uhealth.

[47] Chung J, Brakey HR, Reeder B, Myers O, Demiris G (2023). Community-dwelling older adults’ acceptance of smartwatches for health and location tracking. Int J Older People Nurs.

[48] Li YH, Li YL, Wei MY, Li GY (2024). Innovation and challenges of artificial intelligence technology in personalized healthcare. Sci Rep.

[49] Schwitter N (2022). Social capital in retirement villages: a literature review. Ageing Soc.

[50] (2020). Online nation – 2020 report. https://www.ofcom.org.uk/research-and-data/online-research/online-nation.

[51] Hu X, Xia B, Skitmore M, Buys L, Zuo J (2017). Retirement villages in Australia: a literature review. Pac Rim Prop Res J.

[52] Tan BC, Lau TC, Khan N, Tan WH, Ooi CP (2021). Elderly customers’ open innovation on smart retirement village: what they want and what drive their intention to relocate?. J Open Innov: Technol Mark Complex.

[53] Thunberg S, Johnson E, Ziemke T (2024). Investigating healthcare workers’ technostress when welfare technology is introduced in long-term care facilities. Behav Inf Technol.

