# Supplementary material for: Changing Technology Use, Confidence, and Support Needs Among Older Adults in United Kingdom Retirement Villages: Mixed Methods Study
Source: J Med Internet Res. 2026 Aug 31;28:e92816. doi: 10.2196/92816 (PMC13536979; doi:10.2196/92816)
Supplement: Supplementary file 1 [file jmir-v28-e92816-s001.pdf]

1. About You

1.1 What is your gender?

- ○ Female ○ Male ○ Non-Binary ○ Prefer not to say ○ Other___________

1.2 What is your age group?

○ Under 55	○ 55-59	○ 60-64	○ 65-69	○ 70-74
○ 75-79	○ 80-84	○ 85-89	○ Over 90	○ Prefer not to say

1.3 What is your relationship status?

- ○ Single
- ○ Married or cohabiting with a partner
- ○ With a partner, but not cohabiting
- ○ Widowed
- ○ Prefer not to say
- ○ Other__________________________

1.4 How would you describe your ethnicity?

- ○ White – British
- ○ White – Irish
- ○ White – Gypsy or Irish Traveller
- ○ White – Roma
- ○ White – Other
- ○ Mixed – White and Black Caribbean
- ○ Mixed – White and Black African
- ○ Mixed – White and Asian
- ○ Mixed – Other
- ○ Asian – Indian
- ○ Asian – Pakistani
- ○ Asian – Bangladeshi
- ○ Asian – Chinese
- ○ Asian – Other
- ○ Black – Caribbean
- ○ Black – African
- ○ Black – Other
- ○ Arab
- ○ Prefer not to say
- ○ Other__________________________

1.5 What was, or is, your occupation or career?

____

1.6 What is your highest educational qualification?

- ○ No qualifications
- ○ O-Level or equivalent CSE
- ○ A-Level or equivalent
- ○ Higher education below degree level
- ○ Undergraduate degree
- ○ Postgraduate degree
- ○ Other____

1.7 Do you live on your own?

○ Yes ○ No (Please state who lives with you below)

____

1.8 What is your housing status at Lark Hill?

○ Social renter
○ Temporary renter
○ Shared owner
○ Leaseholder

2. Your Technology Use

2.1 How confident are you with technology such as smart phones, smart TVs and tablets?

○ Not at all confident ○ Not very confident ○ Somewhat confident ○ Fairly confident ○ Very confident

2.2 How have you developed this confidence? *(Select all that apply)*

☐ Self-taught
☐ Training in employment
☐ Training at the village
☐ Learnt from friends or family
☐ Other ______________________________

2.3 How much do you enjoy using technology?

○ Dislike a lot ○ Dislike a little ○ Neither like or dislike ○ Like a little ○ Like a lot

2.4 How often do you send text messages?

○ Every day
○ Several times a week
○ Once a week
○ Once a month
○ Less than once a month
○ Never

2.5 Do you have broadband internet or wi-fi in your home?

○ Yes ○ No ○ Don’t know

2.6 Which of the following devices do you use? ***(Select all that apply)***

- ☐ Computer of laptop at home
- ☐ Mobile phone
- ☐ Digital assistant (e.g. Alexa, Google Home)
- ☐ Tablet (e.g. iPad)
- ☐ Computer in the IT Suite or Library
- ☐ Kindle or other e-reader
- ☐ Other____

2.7 Which devices do you use to connect to the internet? ***(Select all that apply)***

- ☐ Computer of laptop at home
- ☐ Mobile phone
- ☐ Digital assistant (e.g. Alexa, Google Home)
- ☐ Tablet (e.g. iPad)
- ☐ Computer in the IT Suite or Library
- ☐ Kindle or other e-reader
- ☐ Other____

2.8 Do you use the internet for any of the following? ***(Select all that apply)***

- ☐ Online banking
- ☐ Online shopping
- ☐ Keeping in touch (e.g. email, Skype, Zoom, Facebook etc)
- ☐ Finding information
- ☐ Playing or downloading music, TV or films
- ☐ Playing or downloading games
- ☐ None of the above
- ☐ Other__________________

2.9 How do you prefer to contact organisations when you want to get in touch with them? ***(Select one or two options below)***

- ☐ Email
- ☐ Social media (e.g. Facebook or Twitter)
- ☐ Phone
- ☐ Letter
- ☐ Other____

2.10 How often do you use the computers or IT Suite in your village?

- ○ Every day
- ○ Several times a week
- ○ Once a week
- ○ Once a month
- ○ Less than once a month
- ○ Never

2.11 Do you consider that you have an understanding of what a smart device is?

○ Yes ○ No

2.12 Would you consider yourself to be open to using new smart technology?

○ Yes ○ No

2.13 If no, why not?

____

____

____
